# Biofunctional soy-based sourdough for improved rheological properties during storage

**DOI:** 10.1038/s41598-022-22551-z

**Published:** 2022-10-20

**Authors:** Bernadette-Emőke Teleky, Gheorghe Adrian Martău, Floricuța Ranga, Ioana Delia Pop, Dan Cristian Vodnar

**Affiliations:** 1 Institute of Life Sciences, University of Agricultural Sciences and Veterinary Medicine, Calea Mănăştur 3-5, 400372 Cluj-Napoca, Romania; 2 Faculty of Food Science and Technology, University of Agricultural Sciences and Veterinary Medicine, Calea Mănăştur 3-5, 400372 Cluj-Napoca, Romania; 3 Department of Exact Sciences, Horticulture Faculty, University of Agricultural Sciences and Veterinary Medicine, Cluj-Napoca, Calea Mănăştur 3-5, 400372 Cluj-Napoca, Romania

**Keywords:** Biochemistry, Biotechnology

## Abstract

Frozen dough storage, along with its thawing process, negatively affects the quality of the final product. Thus, fermentation with selected cultures and the enrichment of wheat-based dough using a specific soy powder could optimize the viscoelastic quality of frozen dough and increase its nutritional characteristics. Based on these aspects, the present study’s objective was to examine the effects of soy powder addition to wheat flour with single cultures of *Fructilactobacillus florum* DSM 22689 or *Saccharomyces cerevisiae* and coculture with these two microorganisms for 72 h of fermentation. Additionally, the fermentation process was monitored, and viscoelastic behavior and physical–chemical analyses of the fermented sourdough before and after frozen storage were assessed, as soy protein has been proposed to hinder water migration throughout frozen storage. As observed, soy powder, an essential functional ingredient, had a favorable impact on the water-starch-gluten system, and enhanced the viscoelastic behavior before and after 4 weeks of frozen storage.

## Introduction

The use of sourdough in bread production, with a history exceeding 5000 years, has several beneficial effects on the nutritional, sensorial, shelf life, functional, and rheological features of bakery products^1, 2^. Dough, a mixture of flour and water, encompasses a continuous network of protein matrices where each cell is entrained and provides the viscoelasticity needed for qualitative bread production^3^. Wheat flour (WF) possesses particular processability features during dough production due to the development of three-dimensional structures^4^. Gluten, considered the dough frame, is achieved through WF hydration and bestows the quantity and quality of the obtained products^5^.

Baker yeast, which is primarily used for dough leavening through fermentation, produces CO_2_ that is withheld inside the dough’s intricate network, comprising viscoelastic hydrated gluten^6^. Nevertheless, lactic acid bacteria (LAB) are mainly used to improve the organoleptic properties, increase the preservation of the final products and have the main capacity for lactic acid and acetic acid production^7, 8^. Thus, fermentation with LAB and yeasts has been comprehensively studied regarding their abilities to metabolize carbohydrates, lipids, and amino acids, undergo proteolysis, and generate volatile compounds^9^. Organic acids are beneficial to extend shelf life, improve sensory quality^10^, contribute to a higher elasticity and malleability, and increase bread volume^11^.

Rheological assessment of sourdough viscoelastic demeanor is beneficial from the point of view of preparing and processing to ameliorate the bread-making process. In addition, the shelf life of bakery products is usually a couple of days, so consequently, a suitable and economically advantageous alternative is the utilization of frozen dough^12, 13^. However, frozen storage and the subsequent thawing process cause a decline in dough strength, owing to water migration and the formation of ice crystals, which results in a diminished loaf volume^14^. Therefore, several sourdoughs formulations^15–17^ together with different frozen storage conditions^18^, various thawing temperatures^12^ have been thoroughly analyzed to improve the baking performance and quality of the final product^19^.

Whole soy powder (SP) is frequently used in functional food production because diets rich in soy-based foods have several positive health-related effects, such as a low risk of heart disease or distinct age-related diseases^20^. Due to its positive health effects, SP (less than 30%) can be effectively integrated into bakery products and favorably introduced into Westernized diets^21^. A favorable impact of SP-enriched WF during sourdough production is the soy property of increased water holding capacity^22^, which could bypass the negative effects of thawing on dough quality^23^.

Our work presents a novel perspective regarding the utilization of the fructophilic LAB (FLAB) *Fructilactobacillus florum* (*Ff*) during sourdough fermentation on a substrate enriched with SP, which has not been previously studied. Therefore, in the present study, the influence of SP addition to WF for 72 h of fermentation was analyzed, together with its effect on viscosity before and after frozen storage. In addition, the rheological and chemical effects of single and coculture fermentation with *Ff* and *Saccharomyces cerevisiae* (*Sc*) were also investigated, as well as the viability and acidification effects of these microorganisms. The findings of this study will help understanding how SP enrichment and fermentation with select cultures affect the viscoelastic features of sourdough products and whether its inclusion presents added advantages during frozen storage.

## Results

### Viability and pH

**Figure 1:**
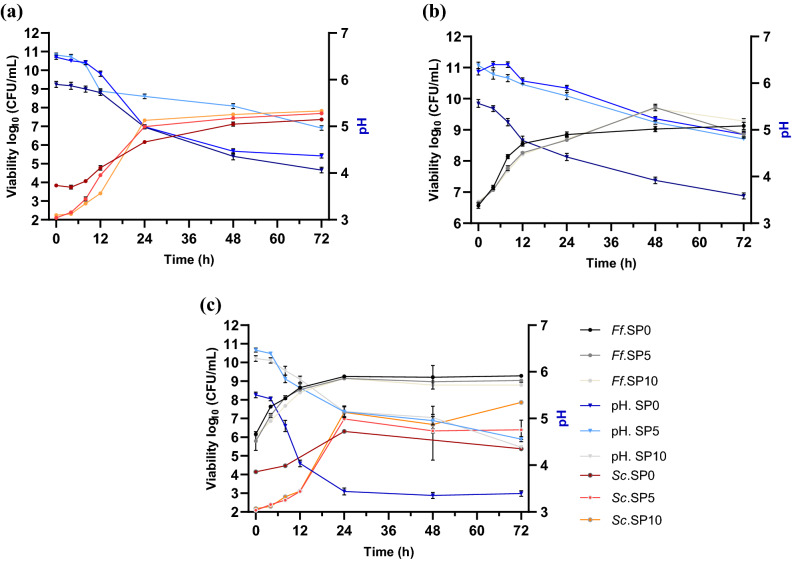
Viable cell counts (red and black lines) and pH profile (blue lines with triangles) of dough fermented with (**a**) *S. cerevisiae* (red lines with squares), (**b**) *F. florum* (black lines with dots), and **c**. *F. florum* + *S. cerevisiae*. Viability and pH values are displayed as the mean values ± SD, log_10_ CFU/mL, *n* = 3, GraphPad Prism Version 8.0.1 (Graph Pad Software, Inc., San Diego, CA, USA); CFU/mL (colony-forming units/milliliter of the sample), SP0—0% soy powder, SP5—5% soy powder, SP10—10% soy powder.

The growth kinetics and acidification for 72 h of fermentation with single and cocultures of *Ff* and *Sc* in different WF and SP mixtures are shown in Fig. 1 and are in agreement with other sourdough evaluations^24, 25^. At the beginning of fermentation, small colony counts with values greater than 4.0 log_10_ CFU/mL for *Sc* and 7.0 log_10_ CFU/mL for *Ff* were exposed and are in line with previous studies^25, 26^. However, the viability of *Ff* and *Sc* in single cultures and cocultures increased in the first 24 h, while only a slight increase occurred afterward. These results indicate that, under the conditions presented in this study, the microorganisms grew between two and three log units throughout the fermentation period in all three batches, reaching final counts of 7.4–7.9 log_10_ CFU/mL with *Sc*, 8.8–9.4 log_10_ CFU/mL with *Ff*, and in the coculture, 8.6–9.4 log_10_ CFU/mL with *Ff*, and 7.4–7.9 log_10_ CFU/mL with *Sc*. In addition, these results are directly correlated with the results from model media fermentation, with values of 9.5–10.7 log_10_ CFU/mL with *Ff*, 7.6–8.4 log_10_ CFU/mL with *Sc*, and in the coculture, 9.2–9.4 log_10_ CFU/mL with *Ff*, and 6.7–7.1 log_10_ CFU/mL with *Sc*. Additionally, coculture fermentation had no considerable negative effect on microorganism viability.

**Figure 2:**
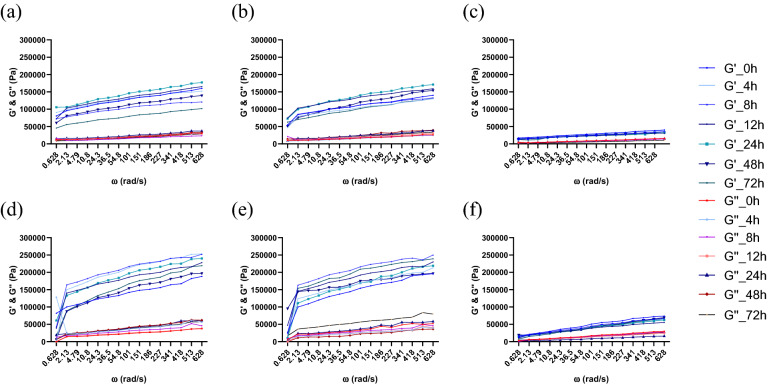
Rheological properties during the fermentation process and frozen storage for *F. florum*. Fermentation influence (**a**) SP10; (**b**) SP5; (**c**) SP0; and after frozen storage (**d**) SP10; (**e**) SP5; (**f**) SP0 (SP0—0% soy powder, SP5—5% soy powder, SP10—10% soy powder).

**Figure 3:**
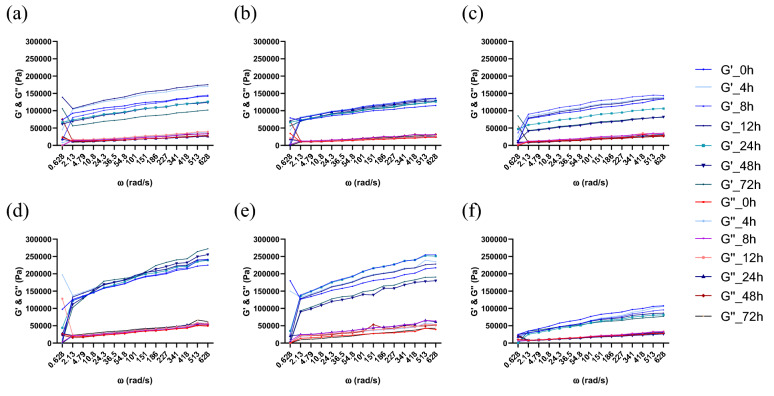
Rheological properties during the fermentation process and frozen storage with *S. cerevisiae.* Fermentation influence (**a**) SP10; (**b**) SP5; (**c**) SP0; and after frozen storage (**d**) SP10; (**e**) SP5; (**f**) SP0 (SP0—0% soy powder, SP5—5% soy powder, SP10—10% soy powder).

**Figure 4:**
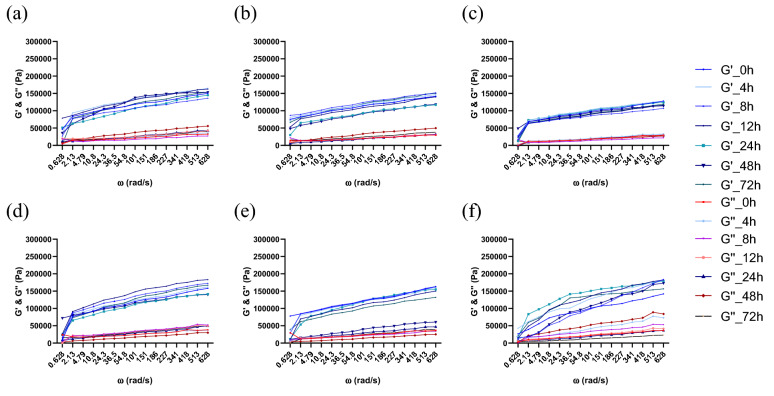
Rheological properties during the fermentation process and frozen storage with the co-cultures of *F. florum* and *S. cerevisiae.* Fermentation influence (**a**) SP10; (**b**) SP5; (**c**) SP0; and after frozen storage (**d**) SP10; (**e**) SP5; (**f**) SP0 (SP0—0% soy powder, SP5—5% soy powder, SP10—10% soy powder).

At time 0, after microorganism inoculation, the pH of each batch was approximately 6.15 ± 0.13, and during the fermentation, a rapid decrease in pH was observed, reaching final values between 4.06 ± 0.06 and 4.98 ± 0.05 in the case of *Sc,* 3.58 ± 0.06 and 4.89 ± 0.08 with *Ff*, and 3.4 ± 0.06 and 4.57 ± 0.05 in the case of the cocultures, as presented in Fig. 1 using mean values from three replications of every batch. This rapid decrease corresponds to those observed in former studies^24, 27^. The most significant decrease was observed in the first 24 h; when the highest increase in viability was seen, the pH did not change significantly (*p* < 0.05), although a slight decrease occurred up to 72 h of fermentation. In the cocultures, the pH of sourdough was the lowest in batch SP0, reaching a final pH value of 3.39 ± 0.06, while in batches SP5 and SP10, the final pH values were 4.57 ± 0.05 and 4.39 ± 0.04, respectively. Additionally, in model media, the pH value decreased from 6.2 to 3.6 with *Ff,* from 6.6 to 4.4 with *Sc,* and from 5.8 to 4.5 in the case of the cocultures. The viscosity decreased when the pH was reduced to values of 4 or less, as observed in batch SP0 and shown in Figs. 2, 3, 4c. In this case, the pH was lower, and consequently, the dough also had a slightly lower viscosity.

### Secondary metabolites characterization

Monosaccharides, such as glucose and fructose, showed a constant decrease in concentration due to fructophilic LAB (FLAB) or yeast consumption (Table 1). In the coculture and single culture with *Sc,* glucose and fructose were consumed after 24–72 h. In the case of *Ff,* the final glucose concentration reached 0.105 ± 0.010 to 0.567 ± 0.080 g/L and that of fructose was 0.045 ± 0.024 to 0.486 ± 0.074 g/L.

**Table 1:** Monosaccharides concentration through fermentation (g/L).

	MO	Time (h)	SP0	SP5	SP10
Glucose	*Sc*	0	1.561 ± 0.050**	0.736 ± 0.070*** (0.583)	0.680 ± 0.075**
		4	1.407 ± 0.040**	0.525 ± 0.024*** (0.874)	0.541 ± 0.057**
		8	0.745 ± 0.048*	0.487 ± 0.074*** (0.753)	0.544 ± 0.146*** (0.102)
		12	0.037 ± 0.064**	0.488 ± 0.060**	0.228 ± 0.051**
		24	0.018 ± 0.023*** (0.220)	N.S	N.S
		48–72	N.D.	N.D.	N.D.
	*Ff*	0	1.327 ± 0.022**	1.255 ± 0.084**	0.995 ± 0.052**
		4	1.244 ± 0.030**	1.501 ± 0.094**	0.771 ± 0.031**
		8	1.925 ± 0.075**	1.035 ± 0.073**	0.725 ± 0.029**
		12	1.319 ± 0.054**	0.661 ± 0.066*** (0.110)	0.549 ± 0.047**
		24	1.518 ± 0.079**	0.706 ± 0.065**	0.396 ± 0.062**
		48	0.910 ± 0.067*	0.694 ± 0.106**	0.312 ± 0.034**
		72	0.432 ± 0.055**	0.567 ± 0.080**	0.105 ± 0.010**
	*Sc* + *Ff*	0	1.025 ± 0.042*	1.825 ± 0.038**	0.498 ± 0.025**
		4	1.628 ± 0.051**	0.896 ± 0.089**	0.393 ± 0.027**
		8	1.118 ± 0.020**	0.559 ± 0.036**	0.070 ± 0.009**
		12	0.719 ± 0.057**	0.333 ± 0.031**	0.028 ± 0.015**
		24	0.036 ± 0.011**	N.D.	N.D.
		48	0.031 ± 0.005**	N.D.	N.D.
		72	N.D.	N.D.	N.D.
Fructose	*Sc*	0	0.910 ± 0.048**	0.618 ± 0.047*** (0.473)	0.670 ± 0.059**
		4	1.275 ± 0.065**	0.796 ± 0.063*** (0.333)	0.886 ± 0.084**
		8	1.235 ± 0.060**	1.637 ± 0.042**	0.843 ± 0.057**
		12	1.833 ± 0.059**	1.306 ± 0.064**	0.733 ± 0.051**
		24	1.408 ± 0.047**	0.482 ± 0.047**	N.D.
		48	N.D.	0.154 ± 0.033**	N.D.
		72	N.D.	N.D.	N.D.
	*Ff*	0	1.915 ± 0.105**	1.063 ± 0.056*	0.824 ± 0.029**
		4	1.292 ± 0.074**	1.082 ± 0.040**	0.359 ± 0.038**
		8	1.859 ± 0.074**	0.777 ± 0.033**	0.271 ± 0.065**
		12	1.766 ± 0.098**	0.272 ± 0.033*** (0.336)	0.196 ± 0.010**
		24	1.163 ± 0.073**	0.421 ± 0.051**	0.110 ± 0.038**
		48	0.512 ± 0.017*** (0.390)	0.554 ± 0.052**	0.061 ± 0.032**
		72	0.156 ± 0.057**	0.486 ± 0.074**	0.045 ± 0.024*** (0.110)
	*Sc* + *Ff*	0	1.199 ± 0.086*** (0.849)	1.164 ± 0.080*	0.944 ± 0.077*
		4	2.195 ± 0.087**	1.433 ± 0.054**	1.013 ± 0.024**
		8	1.872 ± 0.057**	1.333 ± 0.094**	0.895 ± 0.067**
		12	2.014 ± 0.100**	1.309 ± 0.061**	0.406 ± 0.054**
		24–72	N.D.	N.D.	N.D.

Results (displayed as mean values ± SD, g/L, *n* = 3). Data normality was investigated applying the Shapiro-Whilk test. For values, *p* > 0.05, denote normally distributed data (mean ± S.D.). To establish the significant differences between Batch SP0, SP5, and SP10, one-way ANOVA, and post-hoc Tukey HSD tests were applied at every moment for every compound. Suppose the F value was *p* < 0.05, the assessment was carried on, and the significance of differences between means for two batches (SP0 *vs.* SP5; SP5 *vs.* SP10; and SP0 *vs.* SP10) was attained. The Tuckey HSD p-value was introduced in brackets if the *p* > 0.05 and the symbols for the Tuckey HSD interference are as follows: **p < 0.01, *p < 0.05, ***p > 0.05, N.D.—not detected. SP0—0% soy powder, SP5—5% soy powder, SP10—10% soy powder, *Sc*—*Saccharomyces cerevisiae, Ff*—*Fructilactobacillus florum.*

The maltose concentration at the beginning of fermentation was between 1.959 ± 0.054 and 3.718 ± 0.036 g/L, 0.871 ± 0.070 and 1.825 ± 0.046 g/L, and 0.633 ± 0.054 and 0.771 ± 0.035 g/L in SP10, SP5, and SP0, respectively. After 24 h with *Ff*, a slight increase was observed in SP10 (3.916 ± 0. 044 g/L) and SP5 (2.784 ± 0.060 g/L). In coculture, the maltose concentration increased until 24 h, after which it decreased to 0.424 ± 0.04 g/L in SP10 and 0.186 ± 0.042 g/L in SP5 and ceased to be produced after 48 h in SP0, as observed in a similar study^28^. A fluctuating maltose level was observed with *Sc* fermentation (Supplementary Table 1).

Regarding the resulting organic acids produced in all three batches, lactic acid was established as a dominant product at the end of fermentation (Table 2). The highest quantity resulted after 72 h of fermentation in the cocultures, and with the increase in SP concentration, the lactic acid production also increased: SP0 (1.407 ± 0.016); SP5 (1.705 ± 0.032); and SP10 (2.656 ± 0.096). Acetic acid formation through fermentation with the single *Ff* culture was found in the highest quantity at 72 h (0.889 ± 0.092 g/L, 0.631 ± 0.034 g/L, and 0.115 ± 0.023 g/L in SP0, SP5, and SP10, respectively). In the coculture, acetic acid production was between 0.080 ± 0.020 and 0.261 ± 0.058 g/L, and that in the *Sc* batch was between 0.154 ± 0.038 and 0.310 ± 0.028 g/L.

**Table 2:** Main organic acids concentration through fermentation (reported in g/L).

	MO	Time (h)	SP0	SP5	SP10
Lactic acid	*Sc*	0	N.D.	N.D.	N.D.
		4	N.D.	0.030 ± 0.013**	N.D.
		8	N.D.	0.036 ± 0.009**	N.D.
		12	0.166 ± 0.007**	0.034 ± 0.009**	N.D.
		24	0.333 ± 0.083**	0.865 ± 0.035**	0.127 ± 0.010**
		48	0.335 ± 0.020**	1.380 ± 0.009**	0.144 ± 0.015**
		72	1.260 ± 0.082**	0.955 ± 0.036**	0.643 ± 0.024**
	*Ff*	0	N.D.	N.D.	N.D.
		4	0.049 ± 0.010*** (0.348)	0.034 ± 0.019*** (0.348)	0.061 ± 0.030*** (0.348)
		8	0.136 ± 0.032*** (0.732)	0.124 ± 0.051*** (0.732)	0.156 ± 0.057*** (0.732)
		12	0.167 ± 0.046*** (0.468)	0.205 ± 0.039*	0.323 ± 0.026**
		24	0.331 ± 0.037*** (0.632)	0.297 ± 0.045**	0.541 ± 0.048**
		48	0.225 ± 0.034*	0.433 ± 0.026**	1.072 ± 0.076**
		72	1.521 ± 0.018*	1.407 ± 0.009**	1.347 ± 0.048*
	*Sc* + *Ff*	0	N.D.	N.D.	N.D.
		4	0.106 ± 0.014*** (0.229)	0.078 ± 0.004**	N.D.
		8	0.313 ± 0.019**	0.280 ± 0.047**	0.377 ± 0.036**
		12	0.634 ± 0.040*** (0.899)	0.637 ± 0.038**	0.788 ± 0.037**
		24	2.278 ± 0.097**	1.937 ± 0.043**	0.888 ± 0.033**
		48	3.174 ± 0.098**	1.962 ± 0.072**	1.453 ± 0.050**
		72	2.656 ± 0.096**	1.705 ± 0.032**	1.407 ± 0.016**
Acetic acid	*Sc*	0	N.D.	N.D.	N.D.
		4	N.D.	0.063 ± 0.025*** (0.899)	N.D.
		8	N.D.	0.075 ± 0.030*** (0.899)	N.D.
		12	N.D.	0.067 ± 0.027*** (0.899)	N.D.
		24	N.D.	0.124 ± 0.039**	0.273 ± 0.032**
		48	0.305 ± 0.036*	0.199 ± 0.023*	0.305 ± 0.027*** (0.899)
		72	0.310 ± 0.028**	0.154 ± 0.038*	0.273 ± 0.032*** (0.423)
	*Ff*	0–4	N.D.	N.D.	N.D.
		8	0.100 ± 0.019**	0.051 ± 0.13**	N.D.
		12	0.103 ± 0.032*** (0.243)	0.072 ± 0.015*** (0.101)	0.028 ± 0.010*
		24	0.756 ± 0.061**	0.244 ± 0.041**	0.047 ± 0.018**
		48	0.477 ± 0.059*** (0.709)	0.509 ± 0.057**	0.054 ± 0.009**
		72	0.889 ± 0.092**	0.631 ± 0.034**	0.115 ± 0.023**
	*Sc* + *Ff*	0–12	N.D.	N.D.	N.D.
		24	0.603 ± 0.030**	0.188 ± 0.010**	N.D.
		48	0.323 ± 0.062*	0.147 ± 0.071*	0.006 ± 0.004**
		72	0.261 ± 0.058*** (0.096)	0.147 ± 0.035*** (0.096)	0.080 ± 0.020*** (0.096)

Results (displayed as mean values ± SD, g/L, n = 3). Data normality was investigated applying the Shapiro-Whilk test. For values, *p* > 0.05, denote normally distributed data (mean ± S.D.). To establish the significant differences between Batch SP0, SP5, and SP10 one-way ANOVA and post-hoc Tukey HSD tests were applied at every moment for every compound. Suppose the F value was *p* < 0.05, the assessment was carried on, and the significance of differences between means for two batches (SP0 vs SP5; SP5 vs SP10; and SP0 vs SP10) was attained. The Tuckey HSD p-value was introduced in brackets if the *p* > 0.05 and the symbols for the Tuckey HSD interference are as follows: **p < 0.01, *p < 0.05, ***p > 0.05, N.D.—not detected. SP0—0% soy powder, SP5—5% soy powder, SP10—10% soy powder, *Sc—Saccharomyces cerevisiae, Ff—Fructilactobacillus florum.*

The final citric acid concentration was the lowest where *Ff* was present (Supplementary Table 2). In SP0 at 72 h, a quantity of 0.183 ± 0.047 g/L was found, which completely disappeared in SP5 and SP10 in *Ff* single culture. The final average concentration was 0.078 ± 0.022–0.140 ± 0.054 g/L in every batch in coculture. With *Sc,* the final concentrations were higher and ranged between 0.027 ± 0.011 and 0.268 ± 0.048 g/L, as previously reported^24^.

Ethanol production with *Sc,* a specialized pH-tolerant yeast^8^, in SP0 increased from the beginning of fermentation, reaching the highest concentration at 12 h of 1.192 ± 0.065 g/L. In SP5 and SP10, ethanol production started at 24 and 48 h, respectively. The final ethanol concentration at 72 h in SP5 was 1.384 ± 0.053, and that in SP10 was 1.538 ± 0.060 g/L. The same ethanol production was observed in coculture at 72 h: in SP0—1.159 ± 0.067 g/L, in SP5—0.285 ± 0.058 g/L, and in SP10—0.466 ± 0.062 g/L (Supplementary Table 3).

In this study, erythritol was found after 24–48 h only in both single cultures, but in a low quantity (Supplementary Table 4). In batch SP5, *Ff* produced erythritol at a concentration of 0.068 ± 0.021 g/L at 48 h, and 0.072 ± 0.009 g/L, and in SP10 at 72 h was 0.083 ± 0.010 g/L. Batch SP10 with *Ff* did not produce any polyols. Moreover, *Sc* in SP10 produced a quantity of erythritol of 0.091 ± 0.009 g/L; in SP5 was 0.123 ± 0.010 g/L; and in the batch where no SP was added reached 0.304 ± 0.028 g/L at the end of fermentation (at 72 h).

### Rheology of wheat-soy dough

The results obtained through the dynamic rheological measurements of the three batches SP0, SP5, and SP10, with single and cocultures thorough 72 h of fermentation, before (Figs. 2, 3, 4a–c) and after (Figs. 2, 3, 4d–f) frozen storage, were analyzed at a temperature of 30 °C.

The moduli were higher in the sourdough enriched with 10% SP than that enriched with 5% SP, and higher than the batch where no SP was added. The supplementation of SP into WF and its impact on the essential dynamic rheological properties of the frozen dough are displayed in Figs. 2, 3, 4d–f.

As G′ was higher than G″ through every analyzed frequency range, each fermentation, before and after frozen storage, displayed a preponderance for solid behavior, as indicated by the loss tangent results (tan(δ) < 1). SP0 displayed the lowest moduli through fermentation with *Ff* and the most considerable tan(δ) values, which indicated lower elastic behavior and more viscous (thicker) dough.

SP is an important functional ingredient that positively affects the water-starch-gluten system, which stabilizes, maintains, or even increases the viscoelastic behavior in single and cocultures during sourdough fermentation. Compared with batch SP0, the batches SP10 and SP5 had lower values for G′ and G″ at low frequency, indicating that the modulus values also increased with increasing protein content. The highest G′ and G″ values were obtained in cocultures, namely of 153,400 Pa in SP10, 150,000 Pa in SP5, 119,000 Pa in SP0, 40,220 Pa in SP10, 38,054 Pa in SP5, and 29,198 Pa in SP0, respectively.

## Discussion

Increased attention has been given to the fermentation of soy-based products with LAB, especially studies regarding the taste and flavor of the end products, bacterial growth, improved shelf life of the final product, and the positive effects of SP on frozen dough followed by the thawing process^22, 27, 29, 30^. *Ff*, a member of the phylogenetic group of *L. fructivorans,* is a FLAB that grows better on fructose than glucose, stimulating fermentation in an anaerobic environment. Because this group was recently detected, their specific features have not been entirely investigated^31^, such as through these fermentations, their behavior in these three different substrates and in cocultivation with the yeast *Sc*. Based on the results, *Ff* grew well on all three samples and in coculture with *Sc*, as only a slight viability decrease was observed.

A fermentation period of 72 h was selected based on a study by Tyler et al*.*, where it was stated that with *Ff* erythritol and mannitol were observed after 72 h of cultivation^7^. Erythritol and mannitol are low-calorie sweetener^7^ polyols that can substitute for added sugars in bakery products^32^. For instance, erythritol production has been reported in *Ff* at approximately 0.2 g/L*,* mostly on substrates rich in fructose and eventually under anaerobic conditions^7, 33^; nevertheless, in this study, under aerobic conditions, substrate *Ff* produced a concentration between 0.072 ± 0.009 and 0.083 ± 0.010 g/L erythritol. The selection of these two strains and fermentation period was based on the ability of *Ff* to produce polyols and of *Sc* to be used in sourdough production. The coculture of *Ff* and *Sc* produced a low amount of erythritol in our experiments, but a higher production was observed in the single *Sc* fermentation with the highest concentration of 0.304 ± 0.028 g/L on the substrate without SP addition. Mannitol was not identified in any fermentation batches. Ethanol production was only observed in fermentations with *Sc.* This strain metabolizes mainly glucose, but can also metabolize fructose and other 6-carbon molecules, produces predominantly CO_2_ and ethanol, and grows over a broad pH range^34^. The highest ethanol concentration was observed in the single culture (*Sc*), but with the increase in SP addition, the ethanol concentration decreased from 1. 538 ± 0.060 to 0. 150 ± 0.051 g/L. Therefore, in batch SP10, the ethanol concentration was almost double than in the cocultures (1.159 ± 0.067 g/L).

Comparing the content of carbohydrates in all substrates, an increase in SP decreased the contents of mono- and disaccharides found, consequently having a slight diminishing effect on organic acid production (**p < 0.01). Nevertheless, in every batch, the ability of microorganisms to ferment was revealed by the nearly total consumption of carbohydrates, decreased pH, and increased lactic and acetic acid production^26^. Heterofermentative *Ff* degrades hexoses through the Embden-Meyerhoff-Parnas pathway, where fructose and maltose consumption generally occur, particularly after glucose depletion, as stated by Gänzle et al. in 2007^35^. The formation of antimicrobial compounds, such as lactic and acetic acid, enhances the adaptability and competitiveness of FLAB to particular environments and their survival in sourdough fermentation^9^, and subsequently decreases the pH value of sourdough^26^. In addition to lactic and acetic acid, citric acid was also assessed through fermentation, which possesses antimicrobial activity^36^. Citric acid is usually depleted when FLAB are present with low amounts of carbohydrates; this was visible in the *Ff* single cultures of SP5 and SP10, where the carbohydrates were totally depleted, and in the cocultures, the final value was between 0. 078 ± 0.022 g/L and 0.140 ± 0.054 g/L. The decrease in carbohydrates, organic acid production, and the formation of polyols showed that the addition of SP to WF had no significant negative effects (**p < 0.01) on fermentation and produced lactic acid in relatively high quantities, especially in the case of the cocultures, which showed a good symbiotic effect. Even not statistically significant, the addition of SP influenced the lactic acid production in the following pattern values: 2.656 ± 0.096 g/L to 1.705 ± 0.032 g/L and 1.407 ± 0.016 g/L on substrates SP0, SP5, and SP10, respectively.

Based on the rheological results, the modulus values increased in each case with increasing frequency. Furthermore, the storage modulus (G′) was higher than the loss modulus (G″) in every analyzed dough, which indicates that the sourdough samples were less viscous and more elastic^37^. In addition, rheological measurements were also made throughout the fermentation period to determine how each microorganism affects dough viscosity. The viscosity decreased until the end of fermentation, being most visible in the case of the single cultures. The obtained loss tangent results highlight that SP can act as a hydrocolloid with a strengthening effect on the sourdough structure, resulting in a harder, more viscous dough. As indicated by Yamul and Navarro, with increasing water quantity, the moduli values decrease, leading to enhanced chain flexibility by substituting protein–protein connections with water-protein connections^38^. They also indicated that more prominent granules could increase G′ and G″ and decrease the tan(δ) value, which can also be observed in the present experiments; with the addition of SP, the tan(δ) values decreased. The rise in moduli values can also be attributed to the fact that in SP0, the dough had a more acidic pH, which can be assigned to the alterations occurring between protein and starch molecules, as observed in a recent study^39^.

The results presented in Figs. 2, 3, 4 also demonstrate that the addition of SP increased the viscoelastic properties, in the case of every microorganism, at every fermentation moment. For instance, there was a significant difference (**p < 0.01) between *Ff* single and coculture fermentations, batches of SP10 and SP0, as follows: G′ of 153,400 Pa *vs.* 119,000 Pa in the cocultures and G′ of 101,000 Pa *vs.* 31,851 Pa in the single culture with FLAB. There was also a slight nonsignificant difference with *Sc*, but only after frozen storage. As observed in other studies for these fermentations^40^, the negative impact of frozen storage was counterbalanced through the addition of SP, which affected the dough’s viscoelastic properties, the viability of yeast and the quality of frozen dough. The substrates that contain SP, after frozen storage almost completely maintained the viscoelastic properties of the fresh dough, so the impact was not consistent.

Faubion and Hoseney described that the dynamic viscoelastic behavior and testing of sourdough samples are influenced by several factors, such as the flour quality, water content, pH, temperature, microorganisms, mixing time, and strain amplitudes, indicating that the oscillation frequency affects the G′ and G″ values of WF doughs^41^. Through the rheological measurements, with the increase in the dynamic and storage moduli, the frequency also increased, which suggests an increased viscous demeanor of the samples. The present results are in close agreement with those of Sun et al.^51^. They also reported that dough rheology is modified with changes in the composition and starter culture during fermentation^42, 43^.

Although wheat has good water retention capability, foaming features, and viscoelasticity^44^, dough quality after frozen storage is significantly lower due to the formation of ice crystals and the loss of yeast cells. Several earlier studies have revealed substantial G′ and G″ losses in the gluten/glutenin of sourdough during frozen storage^45, 46^. According to Simmons et al., the addition of specialty flours, such as SP, can prevent water migration during frozen storage, primarily through dough thawing. Since soy protein, particularly gluten protein, enhances the water holding potential and disrupts the dough macromolecular standard packing, it can bind covalently and noncovalently to wheat protein^22^. A similar study showed that SP addition substantially enhanced dough texture through microwave heating due to increased water-binding abilities and a high lipid content^47^.

As concluded, an essential parameter for bread manufacturing and to shelf life expansion through frozen storage, the dough should experience minor modifications for high quality^40^. For instance, Yang et al. proved that wheat starch degraded when the viscosity decreased during frozen storage. Nevertheless, where the viscosity remained unaltered, the final product had better quality and enhanced storage duration^48^. Similar results were reported in other studies with WFs enriched with different special flours (amaranth, mesquite, semolina, rice wholemeal, quinoa wholemeal, winter wheat, etc.), or as a single substrate, and the effects of the special flours on fresh dough or after frozen storage are presented in Supplementary Table 5.

The enhancement of WF with SP has, in addition to functional properties, other positive effects on sourdough during frozen storage, such as water-holding properties. The addition of soy proteins gives multiple rheological benefits, as soy proteins can act much like a hydrocolloid and can be used and applied to improve several sensory and textural characteristics of bakery foods. The dynamic rheological characteristics observed in sourdough enriched with SP through frozen storage might be presented as a dough improver.

Considering the importance of microbial strain stability on sourdough consistency and the final bread characteristics^10^, it is crucial to analyze their effects and control on sourdough stability in a sterile environment. Under acidic conditions, *Ff* presented good endurance and favorable carbohydrate conversion, and therefore, it is a proper strain for bread production^9^. This preliminary research requires confirmation in an actual bakery, also with a nonsterile substrate, and inoculum supplementation to initiate an appropriate sourdough microbiota, which represents a future perspective.

## Methods

### Materials and formulations

Commercial WF was utilized in bread making (Băneasa, type 000) with 10.7% protein, 1.3% dietary fiber, 0.48% ash, and 15.3% moisture content. SP was obtained from ground soybeans (*Glycine max* (L.) Merrill) supplied by the Agricultural Research and Development Center Turda (https://scdaturda.ro/onix/), from the Onix variety (conventional tillage method with 60% vegetable residue as green fertilizer). Three formulations of wheat and soy sourdough were prepared by increasing the partial replacement of WF by SP (0, 5, and 10%) as follows: 0% used as control (SP0), 5% (SP5), or 10% (SP10).

### Strains and culture conditions

The microorganisms used throughout this study were the FLAB *Fructilactobacillus florum* DSM No.: 22689 (*Ff*) and *Saccharomyces cerevisiae* (*Sc*) (active-dry yeast—Pakmaya^®^, Izmir, Turkey) obtained from the University of Agricultural Science and Veterinary Medicine Cluj-Napoca. *Ff* was cultivated in De Man, Rogosa and Sharpe (MRS) broth medium (Merck Co., Darmstadt, Germany) with an additional 5.00 g/L fructose. *Sc* was grown in GPY medium (per liter: glucose, 40.0 g; yeast extract, 5.0 g; and peptone, 5.0 g).

Microorganisms activation occurred in 9 mL of medium (MRS/GPY) inoculated with 1 mL of *Ff* or *Sc* and incubated at 30 °C for a period of 24 h. The activated microorganisms (10 mL) were further propagated in 90 mL of fresh medium (MRS/GPY) and incubated one more time for 24 h. The subsequent culture was centrifuged at 7000 rpm for 10 min at 4 °C (Centrifuge 5810R, Eppendorf, Germany), and the formed pellet was suspended twice in saline solution (0.8% NaCl, Sigma–Aldrich Co., Steinheim, Germany). Each suspension was adjusted at 8 log_10_ colony-forming units per milliliter (CFU/mL) for *Ff* and 6 log_10_ CFU/mL for *Sc*. The *Ff* concentration was established with a NanoDrop 1000 spectrophotometer (NanoDrop Technologies, Wilmington, DE, USA) via optical density measurement at 600 nm (OD600) within values of 0.009 and 0.011, while the *Sc* concentration was established using a Thoma counting chamber (Marienfeld, Germany) and a microscope (Nikon, Japan)^49^.

### Model media cultures

The model media cultures were prepared as presented previously^24, 50^ without any WF and SP addition. After microorganism activation in 450 mL of MRS media, 50 mL of established *Ff* and *Sc* cultures were inoculated at the concentration specified at strains and culture conditions. For single and cocultures, the viability, wet biomass (1 mL centrifuged, supernatant eliminated, remaining pellet measured), and pH were monitored through 72 h of fermentation to oversee microorganism viability and development changes.

### Dough preparation and inoculation with select cultures

Doughs were prepared following the method described previously^24^. The amount of water added was similar to the quantity of flour, with a total yield of 300 g of dough. WF was enriched with SP (0, 5 and 10%) and sterilized in the autoclave for 20 min at 121 °C (Autoclave 4002136, J.P. Selecta, Spain), after which sterilized distilled water was added in a volume of 120 mL for single cultures and 90 mL for cocultures. Before microorganism inoculation, the water and wheat-soy flour mixture were thoroughly homogenized.

All three batches of sourdough were inoculated with 30 mL of *Ff* or *Sc* starter culture. In the cocultures, each microorganism was added separately to the dough matrix in a quantity of 30 mL each. Each sourdough was mixed thoroughly for 1 min and fermented at 30 °C under continuous rotation for 72 h. For viable cell count, pH, HPLC, and rheological measurements, samples were prelevated at 0, 4, 8, 12, 24, 48, and 72 h for each batch.

### pH and viable cell count

pH measurements were carried out by diluting 5 g of sourdough sample in 45 mL of distilled water with continuous homogenization on a magnetic stirrer (IKA^®^, RCT basic, Germany) at 22 °C using digital pH meter (InoLab 7110, Germany). Cell viability was determined by diluting 1 g of sourdough sample in 9 mL of saline solution (onefold). After homogenization, 1 mL was rediluted in another tube with 9 mL of saline solution, and this procedure was repeated (fourfold and sixfold) for both microorganisms until the viability was countable on plates. LAB viability was determined by the pour-plate method, and baker yeast was determined by the spread-plate method^25^.

### Rheological measurements

Dynamic rheological measurements of the fermented dough were carried out with an Anton Paar MCR 72 rheometer (Anton Paar, Graz, Austria) equipped with a Peltier plate-plate system (P-PTD 200/Air) supplied with a temperature controller (T = 30 °C) along with a 50 mm diameter smooth parallel plate geometry (PP-50-67300). Initially, fresh sourdough (approximately 3 g) was positioned on the center of the lower plate of the Peltier plate-plate system at a gap of 1 mm between plates and left to rest for 5 min. Following sample supplying, the dough surplus was trimmed, and through the addition of a silicon oil sample, drying was avoided. To determine the dynamic storage (or elastic) modulus (G′, Pa) and loss (or viscous) modulus (G″, Pa), oscillatory frequency sweep tests were performed at an angular frequency (ω) set with a logarithmic ramp and measured amid the intervals of 0.628–628 rad/s^51^. G′ and G″ illustrate the materials’ competence to store the elastic deformation energy, which coincides with the viscous portion of the materials. Additionally, the loss tangent was calculated based on the results obtained for G′ and G″ (tanδ = G″/G′)^52^.

After 4 weeks, frozen samples stored at − 20 °C were thawed at ambient temperature and reanalyzed to evaluate the effects of frozen storage on sourdough viscoelastic behavior^53^.

### High-performance liquid chromatography

The sugar levels, monosaccharide (glucose), and disaccharide (maltose and sucrose) levels, together with the fermentation end products (lactic acid, citric acid, acetic acid, ethanol, and erythritol), were assessed and quantified with the help of HPLC. The depletion or generation of organic acids and additional secondary metabolites was assessed with HPLC-RID (Agilent 1200 series, Santa Clara, CA, USA), First, the samples were homogenized (dough, 1 g sample + 2 mL of distilled H_2_O), vortexed (30 s), sonicated (15 min), centrifuged (8000 rpm for 10 min), and filtered (0.45 µm pore size Millipore membrane filter). The HPLC had a manual injector connected to a refractive index detector (RID) (Agilent Technologies, Santa Clara, CA, USA), solvent degasser, and quaternary pump. After the injection (20 µL of compound), separation occurred on a Polaris Hi–Plex H column, 300 × 7.7 mm (Agilent Technologies, CA, USA), utilizing a 5 mM H_2_SO_4_ as the mobile phase, 80 °C column temperature, sample flow rate of 0.6 mL/min, and 35 °C RID temperature. Then, compound elution took place for 25 min. The acquisition and analysis of the data were prepared utilizing the OpenLab software ChemStation (Agilent Technologies, CA, USA). Through fermentation, sugars (glucose, maltose, fructose), organic acids (lactic acid, citric acid, acetic acid), polyols (1,3 propanediol, glycerol, erythritol, mannitol), and ethanol were identified^54^.

### Statistical analysis

Statistical analysis of the viability and pH were carried out with the mean values ± standard deviations (SDs) (performed in triplicate) utilizing Graph Prism Version 8.0.1. (GraphPad Software Inc., San Diego, CA, USA).

Data analysis of the essential chemical variables was performed with IBM SPSS Statistics 19 (IBM, Armonk, NY). Each batch and trial were performed in triplicate, and the results are displayed as the mean values ± SDs. For data normality, the Shapiro–Wilkinson test was applied^55^. Statistically significant differences in means were considered at *p* < 0.05. To determine significant differences between each substrate (SP0, SP5, SP10), a one-way ANOVA test and a post hoc Tukey HSD test were applied at every moment for each single organic acid, sugar, or polyol^56^. In the case of obtaining for the F value *p* < 0.05, the calculations were carried out, and the significance of differences between the means for two-two batches was obtained. If the obtained F value was *p* > 0.05, the results would display no significant difference between batches. Other statistical analyses were also considered to consolidate the results, such as the Holm–Bonferroni method and the post hoc Scheffé test. In preponderance, the same significations were obtained, as in the case of Tukey’s test. Subsequently, the following symbols were used: **p < 0.01, *p < 0.05, NS *p* > 0.05.

## Supplementary Information

Supplementary Information.

## Data Availability

All data used during the current study are included in this published article or are available from the corresponding author on reasonable request.
