# Supplementary material for: Biofunctional soy-based sourdough for improved rheological properties during storage
Source: Sci Rep. 2022 Oct 20;12:17535. doi: 10.1038/s41598-022-22551-z (PMC9584935; doi:10.1038/s41598-022-22551-z)
Supplement: Supplementary file 1 — Supplementary Information. [file 41598_2022_22551_MOESM1_ESM.pdf]

Biofunctional soy-based sourdough for improved rheological properties during storage

Authors: Bernadette-Emőke Teleky[1], Gheorghe Adrian Martău[1,2], Floricuța Ranga[2], Ioana Delia Pop[3], and Dan Cristian Vodnar[1,2,*]

[1] Institute of Life Sciences, University of Agricultural Sciences and Veterinary Medicine, Calea Mănăştur 3-5, 400372 Cluj-Napoca, Romania; adrian.martau@usamvcluj.ro (GAM.); bernadette.teleky@usamvcluj.ro (BET.)

[2] Faculty of Food Science and Technology, University of Agricultural Sciences and Veterinary Medicine, Calea Mănăştur 3-5, 400372 Cluj-Napoca, Romania; floricutza_ro@yahoo.com (FR)

[3] Department of Exact Sciences, Horticulture Faculty, University of Agricultural Sciences and Veterinary Medicine, Cluj-Napoca, Calea Mănăştur 3-5, 400372 Cluj-Napoca, Romania; popioana@usamvcluj.ro (IDP)

*Correspondence: dan.vodnar@usamvcluj.ro; Tel.: +40747341881

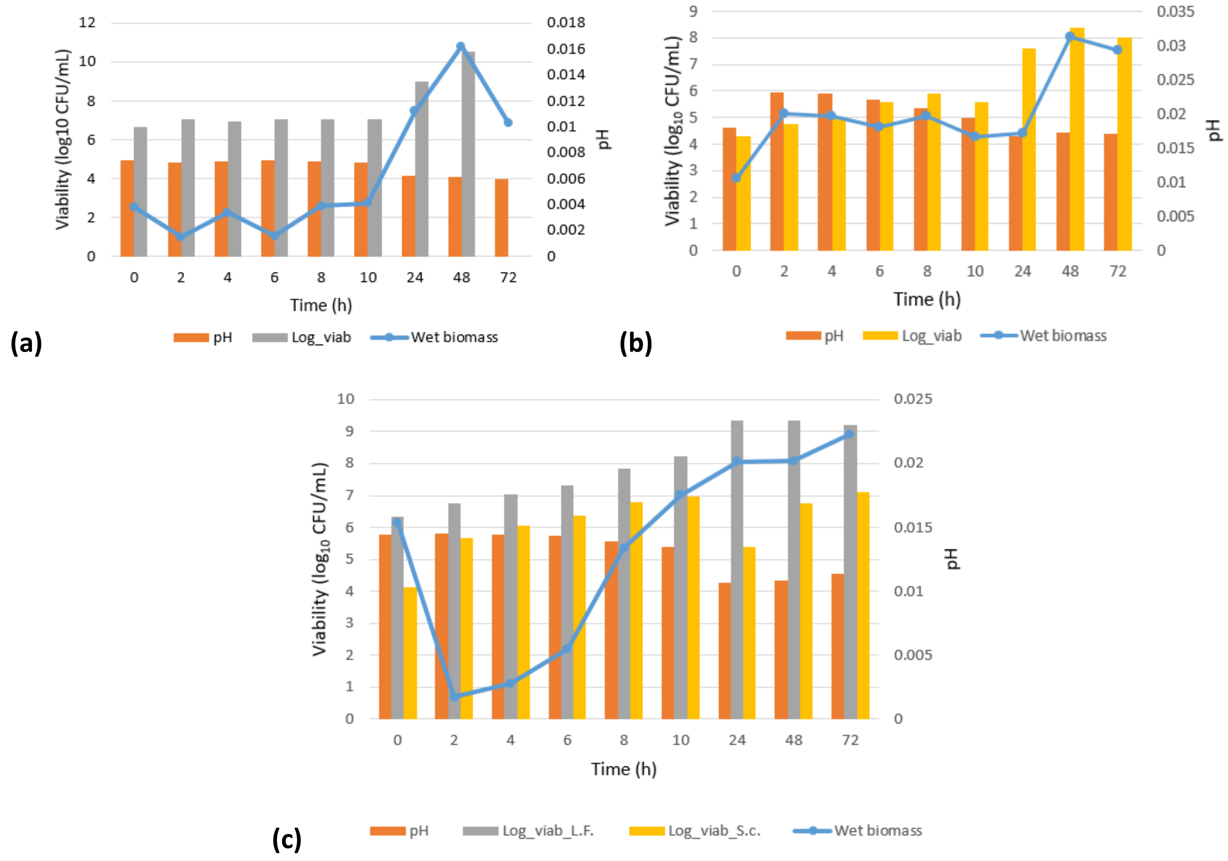

Supplementary Figure 1. Cell viability and pH profile of model media fermented with **a**. *F. florum;* **b**. *S. cerevisiae;* **C**. *F. florum* + *S. cerevisiae*, blue line – wet biomass, orange column – pH, grey column – viability *Ff*, *yellow column* - viability *Sc*.

Supplementary Table 1. Disaccharides concentration through fermentation (g/L)

	MO	Time (h)	SP0	SP5	SP10
Maltose	*Sc*	0	3.718 ± 0.036**	0.871 ± 0.070*** (0.074)	0.734 ± 0.066**
		4	4.624 ± 0.040**	0.950 ± 0.041*	1.081 ± 0.067**
		8	3.272 ± 0.056**	1.790 ± 0.030**	1.162 ± 0.039**
		12	5.258 ± 0.053**	1.472 ± 0.040**	1.235 ± 0.020**
		24	5. 546 ± 0.388**	0.678 ± 0.046*** (0.089)	0.198 ± 0.017**
		48	2.489 ± 0.040**	0.332 ± 0.016**	0.116 ± 0.015**
		72	3.217 ± 0.031**	2.205 ± 0.021**	0.137 ± 0.035**
	Lf	0	3.439 ± 0.033**	1.825 ± 0.046**	0.633 ± 0.054**
		4	3.719 ± 0.030**	1.713 ± 0.063**	0.888 ± 0.029**
		8	2.177 ± 0.038**	1.030 ± 0.068**	1.421 ± 0.103**
		12	1.885 ± 0.086**	1.107 ± 0.138*** (0.899)	1.081 ± 0.050**
		24	3.916 ± 0. 044**	2.784 ± 0.060**	0.319 ± 0.064**
		48	3.867 ± 0.089*	4.156 ± 0.121**	N.D.
		72	3.361 ± 0.058**	4.874 ± 0.049**	N.D.
	Sc + Lf	0	1.959 ± 0.054**	1.685 ± 0.062**	0.771 ± 0.035**
		4	3.289 ± 0.051**	1.946 ± 0.080**	0.751 ± 0.058**
		8	3.109 ± 0.068**	1.883 ± 0.053**	0.504 ± 0.061**
		12	4.542 ± 0.102**	2.980 ± 0.073**	0.322 ± 0.029**
		24	1.958 ± 0. 067**	1.179 ± 0.090**	0.093 ± 0.055**
		48	0.486 ± 0.068**	0.221 ± 0.025**	N.D.
		72	0.424 ± 0.048**	0.186 ± 0.042**	N.D.

Results (displayed as mean values ± SD, g/L, n = 3). Data normality was investigated applying the Shapiro-Whilk test. For values, $p>0.05$, denote normally distributed data (mean ± S.D.). To establish the significant differences between Batch SP0, SP5, and SP10 one-way ANOVA test and post-hoc Tukey HSD test were applied at every moment for every compound. Suppose the F value was $p<0.05$, assessment was carried on, and the significance of differences between means for two batches (SP0 vs SP5; SP5 vs SP10; and SP0 vs SP10) was attained. The Tuckey HSD p-value was introduced in brackets if the $p>0.05$ and the symbols for the Tuckey HSD interference are as follows: ** $p<0.01$, * $p<0.05$, *** $p>0.05$, N.D. – not detected. SP0 – 0% soy flour, SP5 – 5% soy flour, SP10 – 10% soy flour, *Sc* – *Saccharomices cerevisiae, Lf – Fructilactobacillus florum.*

Supplementary Table 2. Citric acid concentration through fermentation (g/L)

	MO	Time (h)	SP0	SP5	SP10
**Citric acid**	***S. cerevisiae***	0	0.666 ± 0.043**	0.346 ± 0.064**	0.035 ± 0.026**
		4	0.534 ± 0.052**	0.146 ± 0.074*** (0.101)	0.032 ± 0.029**
		8	0.343 ± 0.056**	0.173 ± 0.040*	0.041 ± 0.017**
		12	0.422 ± 0.025**	0.187 ± 0.055**	0.039 ± 0.017**
		24	0.802 ± 0.051**	0.168 ± 0.057*	0.030 ± 0.013**
		48	0.154 ± 0.036*** (0.250)	0.222 ± 0.070**	0.028 ± 0.017*
		72	0.169 ± 0.046*	0.268 ± 0.048**	0.027 ± 0.011**
	L. florum	0	0.964 ± 0.061**	0.320 ± 0.026**	0.026 ± 0.007**
		4	0.866 ± 0.069**	0.487 ± 0.039**	0.018 ± 0.003**
		8	0.780 ± 0.042**	0.431 ± 0.025**	0.060 ± 0.008**
		12	0.566 ± 0.040**	0.048 ± 0.013*** (0.278)	0.014 ± 0.002**
		24	0.436 ± 0.040**	N.D.	N.D.
		48	0.173 ± 0.045**	N.D.	N.D.
		72	0.183 ± 0.047**	N.D.	N.D.
	S. cerevisiae + L. florum	0	0.333 ± 0.051*** (0.148)	0.198 ± 0.026*** (0.148)	0.222 ± 0.024*** (0.148)
		4	0.507 ± 0.032**	0.241 ± 0.014*** (0.608)	0.193 ± 0.014**
		8	0.492 ± 0.028**	0.233 ± 0.016*** (0.365)	0.170 ± 0.017**
		12	0.472 ± 0.036**	0.240 ± 0.038*** (0.342)	0.180 ± 0.011**
		24	0.229 ± 0.027**	0.055 ± 0.005*	0.171 ± 0.033*** (0.260)
		48	0.131 ± 0.067*** (0.326)	0.075 ± 0.017*	0.194 ± 0.022*** (0.257)
		72	0.140 ± 0.054*** (0.172)	0.078 ± 0.022*** (0.172)	0.107 ± 0.014*** (0.172)

Results (displayed as mean values ± SD, g/L, n = 3). Data normality was investigated applying the Shapiro-Whilk test. For values, $p>0.05$, denote normally distributed data (mean ± S.D.). To establish the significant differences between Batch SP0, SP5, and SP10 one-way ANOVA test and post-hoc Tukey HSD test were applied at every moment for every compound. Suppose the F value was $p<0.05$, assessment was carried on, and the significance of differences between means for two batches (SP0 vs SP5; SP5 vs SP10; and SP0 vs SP10) was attained. The Tuckey HSD p-value was introduced in brackets if the p>0.05 and the symbols for the Tuckey HSD interference are as follows: **$p<0.01$, *$p<0.05$, ***$p>0.05$, N.D. – not detected. SP0 – 0% soy flour, SP5 – 5% soy flour, SP10 – 10% soy flour, *Sc – Saccharomices cerevisiae, Lf – Fructilactobacillus florum.*

Supplementary Table 3. Ethanol concentration through fermentation (g/L)

	MO	Time (h)	SP0	SP5	SP10
Ethanol	***S. cerevisiae***	0	N.D.	N.D.	0.651 ± 0.012**
		4	N.D.	N.D.	1.029 ± 0.136**
		8	N.D.	N.D.	1.172 ± 0.027**
		12	N.D.	N.D.	1.192 ± 0.065**
		24	N.D.	0.030 ± 0.011**	0.196 ± 0.036**
		48	1.252 ± 0.060**	0.569 ± 0.055**	0.127 ± 0.014**
		72	1.538 ± 0.060*	1.384 ± 0.053**	0.150 ± 0.051**
	S. cerevisiae* + *L. florum	0-4	N.D.	N.D.	N.D.
		8	N.D.	N.D.	0.686 ± 0.065**
		12	N.D.	N.D.	1.884 ± 0.061**
		24	0.133 ± 0.044*** (0.586)	0.016 ± 0.011**	2.308 ± 0.147**
		48	0.433 ± 0.058*** (0.309)	0.347 ± 0.026**	1.753 ± 0.079**
		72	0.466 ± 0.062*	0.285 ± 0.058**	1.159 ± 0.067**

Results (displayed as mean values ± SD, g/L, n = 3). Data normality was investigated applying the Shapiro-Whilk test. For values, $p>0.05$, denote normally distributed data (mean ± S.D.). To establish the significant differences between Batch SP0, SP5, and SP10 one-way ANOVA test and post-hoc Tukey HSD test were applied at every moment for every compound. Suppose the F value was $p<0.05$, assessment was carried on, and the significance of differences between means for two batches (SP0 vs SP5; SP5 vs SP10; and SP0 vs SP10) was attained. The Tuckey HSD p-value was introduced in brackets if the $p>0.05$ and the symbols for the Tuckey HSD interference are as follows: **$p<0.01$, *$p<0.05$, ***$p>0.05$, N.D. – not detected. SP0 – 0% soy flour, SP5 – 5% soy flour, SP10 – 10% soy flour, *Sc* – *Saccharomices cerevisiae*, *Lf* – *Fructilactobacillus florum.*

Supplementary Table 4. Erythritol concentration through fermentation (g/L)

	MO	Time (h)	SP0	SP5	SP10
**Erythritol**	***S. cerevisiae***	0-12	N.D.	N.D.	N.D.
		24	N.D.	0.068 ± 0.005*** (0.495)	0.066 ± 0.007**
		48	0.061 ± 0.008*** (0.53)	0.115 ± 0.019*** (0.53)	0.107 ± 0.017*** (0.53)
		72	0.304 ± 0.028**	0.123 ± 0.010*** (0.899)	0.091 ± 0.009**
	L. florum	0-24	N.D.	N.D.	N.D.
		48	N.D.	0.068 ± 0.021*** (0.899)	N.D.
		72	N.D.	0.072 ± 0.009**	0.083 ± 0.010*** (0.516)

Results (displayed as mean values ± SD, g/L, n = 3). Data normality was investigated applying the Shapiro-Whilk test. For values, p>0.05, denote normally distributed data (mean ± S.D.). To establish the significant differences between Batch SP0, SP5, and SP10 one-way ANOVA test and post-hoc Tukey HSD test were applied at every moment for every compound. Suppose the F value was $p<0.05$, assessment was carried on, and the significance of differences between means for two batches (SP0 vs SP5; SP5 vs SP10; and SP0 vs SP10) was attained. The Tuckey HSD p-value was introduced in brackets if the $p>0.05$ and the symbols for the Tuckey HSD interference are as follows: **$p<0.01$, *$p<0.05$, ***$p>0.05$, N.D. – not detected. SP0 – 0% soy flour, SP5 – 5% soy flour, SP10 – 10% soy flour, *Sc – Saccharomices cerevisiae, Lf – Fructilactobacillus florum.*

Supplementary Table 5. Dynamic rheological measurements in similar studies on fresh dough and after frozen storage

Substrate	Microorganism	G' (Pa)	G'' (Pa)	Frequency (Hz) ω (rad/s)	T (℃)	Reference
Whole WF	*Lb. plantarum* LB-1<br>*Lb. plantarum* F-50<br>*Lb. plantarum* F-3	~1650000<br>~900000<br>~135000	~600000<br>~380000<br>~525000	40 Hz	25 ± 1	1
WF+5% Am.<br>WF+25% Am.<br>WF+5% GAm.<br>WF+25% GAm.	N.D.<br>N.D.<br>N.D.<br>N.D.	21700<br>23500<br>21200<br>21300	7900<br>9100<br>8800<br>9000	1 Hz	25 ± 0.1	2
WF	N.D.	~12500	~10000	0.628 - 628 rad/s	30	3
WF+15%MF<br>WF+15%MF – *FS1*<br>WF+35%MF<br>WF+35%MF – *FS1*	N.D.<br>N.D.<br>N.D.<br>N.D.	~75000<br>~75000<br>~100000<br>~80000	~15000<br>~15000<br>~50000<br>~30000	100 Hz	25	4
Sorghum	*P. pentosaceus* LD7	3.07	6.23	0.1 Hz	30	5
	P. pentosaceus SA8	0.88	2.87			
WW – *FS2*<br>WW – *FS3*<br>WW – *FS4*<br>WW – *FS5*<br>WW – *FS6*<br>WW – *FS7*	N.D.<br>N.D.<br>N.D.<br>N.D.<br>N.D.<br>N.D.	~25000<br>~20000<br>~17500<br>~16000<br>~15000<br>~12500	~13000<br>~11000<br>~10000<br>~9000<br>~7000<br>~6000	100 rad/s	25	6
SF - MOD	N.D.	~36000	~12000	0.1 – 10 Hz	20	7
SF - OLD	N.D.	~32000	~12000			
QWF<br>QWF - FS<br>RWF<br>RWF - FS	*Lb. plantarum* ATCC 8014	~10000<br>~25000<br>~20000<br>~25000	~5000<br>~8000<br>~7000<br>~8000	0.628 - 628 rad/s	30	8
WF + EPS<br>WF - EPS	*Lb. curvatus* 69B2	~8000<br>~5000	~2000<br>~4000	1- 100 rad/s	25	9
WF – Ferm.25 ℃ 0 h<br>WF – Ferm.25 ℃ 72 h<br>WF – Ferm.35 ℃ 0 h<br>WF – Ferm.35 ℃ 72 h	MSC	~6500<br>~1000<br>~0<br>~800	~3000<br>~500<br>~3000<br>~500	0.1 – 20 Hz	20	10

The highest loss (G') and storage modulus (G") values were included only, at the highest frequency (Hz) or angular frequency (rad/s). ω - Angular frequency, WF – wheat flour, Am. – amaranth, GAm. – Germinated Amaranth, MF – mesquite flour, MaF – maize flour, WW – winter weath, SF – semolina flour, MOD – modern cultivar, OLD – old genotype, QWF- quinoa wholemeal flour, RWF - rice wholemeal flour, Ferm. - fermented, MSC – multiple strain culture, *FS1* – frozen storage for 90 days at -20 ℃, WW – *FS2* - frozen storage for 1 day at -40 ℃, WW – *FS3* - frozen storage for 1 day at -30 ℃, WW – *FS4* - frozen storage for 1 day at -20 ℃, WW – *FS5* - frozen storage for 30 days at -40 ℃, WW – *FS6* - frozen storage for 30 days at -30 ℃, WW – *FS7* - frozen storage for 30 days at -20 ℃,

References

1. Sun, L. *et al.* A novel lactic acid bacterium for improving the quality and shelf life of whole wheat bread. *Food Control* **109**, 106914 (2020).

2. Guardianelli, L. M., Salinas, M. V. & Puppo, M. C. Hydration and rheological properties of amaranth-wheat flour dough: Influence of germination of amaranth seeds. *Food Hydrocoll.* **97**, 105242 (2019).

3. Sun, X., Koksel, F., Nickerson, M. T. & Scanlon, M. G. Modeling the viscoelastic behavior of wheat flour dough prepared from a wide range of formulations. *Food Hydrocoll.* **98**, 105129 (2020).

4. Bigne, F., Ferrero, C. & Puppo, M. C. Effect of freezing and frozen storage on mesquite – wheat dough for panettone - like breads. *J. Food Meas. Charact.* 1–9 (2019) doi:10.1007/s11694-019-00206-4.

5. Olojede, A. O., Sanni, A. I. & Banwo, K. Rheological, textural and nutritional properties of gluten-free sourdough made with functionally important lactic acid bacteria and yeast from Nigerian sorghum. *Lwt* **120**, 108875 (2020).

6. Yang, J. *et al.* Effect of freezing rate and frozen storage on the rheological properties and protein structure of non-fermented doughs. *J. Food Eng.* **293**, 110377 (2021).

7. Farbo, M. G. *et al.* Improving the quality of dough obtained with old durum wheat using hydrocolloids. *Food Hydrocoll.* **101**, 105467 (2020).

8. Chiş, M. S. *et al.* Quinoa sourdough fermented with lactobacillus plantarum ATCC 8014 designed for gluten-free muffins—a powerful tool to enhance bioactive compounds. *Appl. Sci.* **10**, 1–23 (2020).

9. Palomba, S. *et al.* Polyphasic screening, homopolysaccharide composition, and viscoelastic behavior of wheat sourdough from a Leuconostoc lactis and Lactobacillus curvatus exopolysaccharide-producing starter culture. *Appl. Environ. Microbiol.* **78**, 2737–2747 (2012).

10. Casado, A. *et al.* Effect of fermentation on microbiological, physicochemical and physical characteristics of sourdough and impact of its use on bread quality. *Czech J. Food Sci.* **35**, 496–506 (2017).
